# Unsupervised Learning Composite Network to Reduce Training Cost of Deep Learning Model for Colorectal Cancer Diagnosis

**DOI:** 10.1109/JTEHM.2022.3224021

**Published:** 2022-11-21

**Authors:** Jirui Guo, Wuteng Cao, Bairun Nie, Qiyuan Qin

**Affiliations:** Department of Colorectal SurgeryThe Sixth Affiliated Hospital, Sun Yat-sen University Guangzhou 510655 China; Department of RadiologyThe Sixth Affiliated Hospital, Sun Yat-sen University Guangzhou 510655 China; School of Electrical Computer and Telecommunications EngineeringUniversity of Wollongong 8691 Wollongong NSW 2522 Australia

**Keywords:** Artificial intelligence, computer aided diagnosis, deep learning, image filtering, oncology, unsupervised learning

## Abstract

Deep learning facilitates complex medical data analysis and is increasingly being explored in colorectal cancer diagnostics. However, the training cost of the deep learning model limits its real-world medical utility. In this study, we present a composite network that combines deep learning and unsupervised K-means clustering algorithm (RK-net) for automatic processing of medical images. RK-net was more efficient in image refinement compared with manual screening and annotation. The training of a deep learning model for colorectal cancer diagnosis was accelerated by two times with utilization of RK-net-processed images. Better performance was observed in training loss and accuracy achievement as well. RK-net could be useful to refine medical images of the ever-expanding quantity and assist in subsequent construction of the artificial intelligence model.

## I. Introduction

Colorectal cancer (CRC) is the third most common malignancy worldwide and the second most common cause of cancer-specific death [1]. CRC is a heterogeneous disease wherein accurate determination of biological characteristics for different patients is the key to precision therapy [2], [3]. Artificial intelligence (AI) is a computer technology that mimics human intelligence in learning and problem solving [4]. Machine learning (ML) and deep learning (DL) are AI methods increasingly used to analyze medical data and build predictive models. Substantial progress has been made in these techniques and their applications to CRC diagnostics [5]. Convolutional neural networks (CNNs) are DL methods characterized by consecutive node layers to process structured arrays of data. CNNs are widely utilized for digital image classification and have achieved good performances in the prediction, staging, and prognosis of CRC [6], [7]. Moreover, DL may facilitate the utilization of large data produced by radiologic examinations, such as computed tomography (CT) and magnetic resonance imaging (MRI) [8].

However, developing DL models is difficult and expensive [9]. Obstacles to model construction include powerful hardware, vast data, the time cost and the complexity of training methods [10], [11]. As for medical image analysis, data processing is necessary but laborious. Clinically, target delineation is a crucial step to provide information on the organ shape and volume. Manual separation is the routine approach that is limited by the time consumption and intra/inter-rater variations. Automatic segmentation by networks is challenging, considering the balance of efficiency and reliability. Although supervised learning tasks require abundant data of high-quality, excessive variables at the model input level may complicate the algorithm training and interpretation. Otherwise, variables of interest are hidden behind all available information, where a full model outperforms the subgroups with limited features, especially in the validation of new datasets. In addition, it may take days to weeks to train a neural network for large-scale datasets from scratch, leading to high costs in the research programme.

The accuracy of deep neural networks largely depends on the quality and amount of data. Standardized annotation and reliable data sources are also critical [12]. In general, manual screening and annotation for region of interest (ROI) are fundamental steps for supervised learning in computer vision [13]. However, the procedures are usually time-consuming and cost-intensive [14]. It is reported that some algorithms can assist in clinical images annotation, but the automatic method is particularly challenging in the context of the complicated abdominal anatomy [15]. Furthermore, the annotation of pixel-level for medical images requires professional expertise by experienced radiologists, thus it is laborious to obtain a large-scale labeled dataset of high-quality.

Unsupervised learning is an efficient ML method to identify subgroups within brand-new datasets. It is often used as a preparation step for subsequent tasks to improve the overall feasibility [16]. K-means clustering is an elegant unsupervised learning algorithm. It is suitable for large-scale medical data with advantages in computing speed, cost savings, and minimal disturbance by data outliers [17].

In the present study, we proposed a composite network that combines deep learning and K-means clustering algorithms called RK-net. This network was designed to automatically remove irrelevant images and preserve imaging slices at tumor-level. We aimed to validate the RK-net in processing complex medical images with comparisons to the method of manual screening and annotation, and to test its efficacy in optimizing a DL model for CRC diagnosis.

The manuscript is structured as follows: Section I introduces the AI techniques and their applications in CRC diagnostics; Section II describes the structure of the composite network, data settings, and training method; Section III demonstrates the effect of RK-net on the DL model; Section IV concludes and takes a translational outlook on the results of this study.

## II. Method

### A. Original Material

We identified a cohort of 360 consecutive patients from the prospective database of colorectal cancer at the Sixth Affiliated Hospital of Sun Yat-sen University (SAH-SYSU), Guangzhou, China, a national high-volume colorectal cancer institution. Imaging data were retrospectively extracted and reviewed. All patients were divided into two equal groups based on pathological diagnosis, corresponding to different molecular pathological types. We refer to the patients as Class1 and Class2 for convenience of research. All patients met the following requirements: (1) pathologically diagnosed as colorectal adenocarcinoma; (2) aged ranged from 18-80 years; (3) possessing complete demographic, treatment, and imaging data. The patients who had concurrent malignancy other than colorectal cancer were excluded. Imaging examination data (stored in DICOM format) and clinical data of all enrolled patients were collected. Quality control of the research data was carried out by two experienced clinicians with senior professional titles.

### B. Datasets

We randomly divided the study population to 300 patients as the training dataset and 60 patients as the testing dataset. All data were divided into two categories based on the label. Three processing methods were compared. The proposed RK-net automatically removed irrelevant images and preserved imaging slices at tumour-level. Manual annotation provided segmented images corresponding to the regions of interest (ROIs) as the classification basis. The ROIs of tumour were manually delineated using the ITK-SNAP tool. Manual screening streamlined images at the discretion of experienced radiologists, excluding irrelevant slices from datasets. CT images were converted into DICOM standard format and stored as NII files. Python-OpenCV packages were introduced to split NII files axially.

### C. Platform Building

We constructed a server for data processing and model training. The platform was based on a Standard GPU Server with Xeon E5 2678V3, 32GB DDR4-memory and NVIDIA RTX2070S. According to the NVIDIA’s advice, we selected NVIDIA CUDA Toolkit 10.1 and cuDNN 7.5 to build the compiling environment, and used Anaconda to build the training and testing environment (TensorFlow-GPU 1.14.0, Python 3.6.12). The NVIDIA system management interface was deployed to facilitate the processing.

### D. RK-Net Architecture

The composite network RK-net consists of several parts as follows:

The first part of the composite network is a specially designed medical image processor that performs the batch processing of raw data and converts images into readable forms. Programme components separate individual information and erase personal privacy data.

**FIGURE 1.**
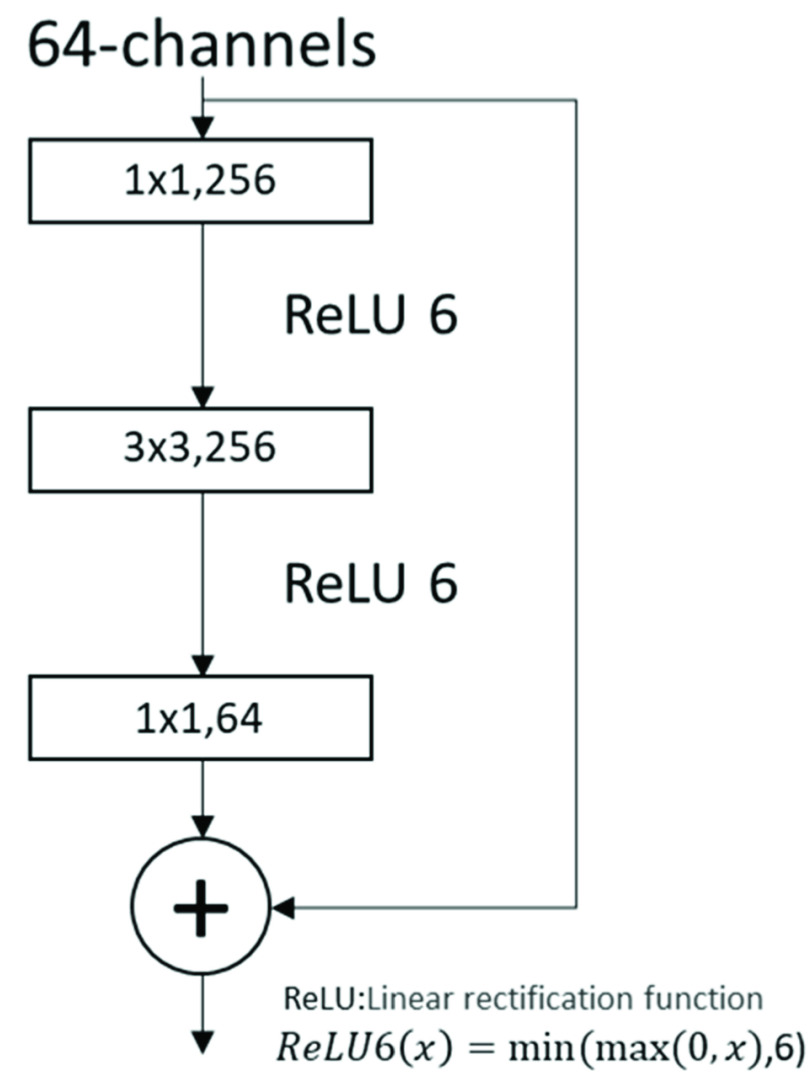
MobileNetV2 special network bottleneck layer structure.

The second part of the composite network is a pre-trained neural network MobileNetV2 that differentiates the transformed images. This neural network is based on an inverted residual structure, in which the shortcut connections are located between the thin bottleneck layers (Fig. 1).

After input of the image information, the matrix is mapped to the higher dimension and then restored to the lower dimension through the convolution layer. The structure finally ensures the correct feature extractions while reducing the computation [18]. MobileNetV2 has been pre-trained and adjusted with parameters on complex public datasets. It performs well in the image classification tasks, achieving a certain level of accuracy with limited model parameters and computation. Moreover, this design of the second part brings in a custom module that can switch between multiple models in accordance with the actual data forms.

The third part of the composite network is the unsupervised classifier with the K-means clustering algorithm, where pre-classification results are introduced for discrimination. The classification principle of the K-means algorithm is based on “ (1) ”, “ (2) ”:

\begin{align*} \mathrm {E}=&\sum \nolimits _{i=1}^{k} \sum \limits _{x\in C_{i}}^{} {\vert \vert x-\mu _{i}\vert \vert }_{2}^{2} \tag{1}\\ \mu _{i}=&\frac {1}{\vert C_{i}\vert }\sum \nolimits _{x\in C_{i}} x\tag{2}\end{align*}

In “ (1) ” and “ (2) ” equations, x is the sample value. k is the number of clustering sample clusters. $C_{i}$ is the division of sample clusters. $\mu _{i}$ is the mean vector of cluster $C_{i}$. E is the algorithm to minimize the square error of clustering. While the smaller the value of E is, the higher sample similarity exists in the cluster [19], [20]. The number of clusters is set to $\text{k}=2$ to remove irrelevant images in the native dataset.

The final results of classification are saved as CSV files. Images are imported to the corresponding folders by the custom setting rules. The last part of RK-net is the image processing module to convert images to the required format. This module is developed based on the OpenCV programme package. The conversion process is documented and indexed by classification results.

**FIGURE 2.**
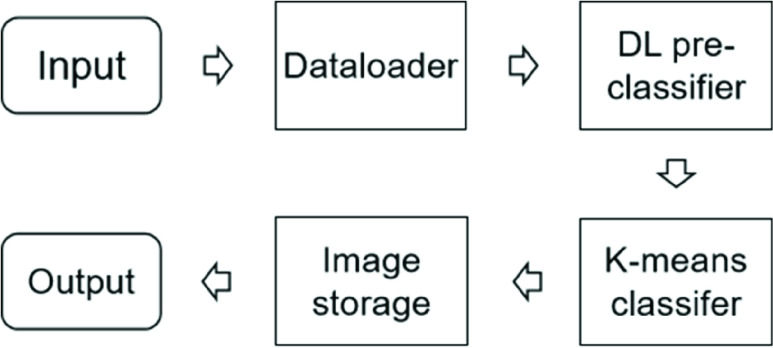
The architecture of RK-net.

The whole architecture of RK-net is shown in Fig 2.

### E. CRC Diagnostic Model

We built a CRC diagnostic model to test the image optimization by the RK-net composite network. The model was based on the pre-trained ResNet-101 network. We used the stochastic gradient descent (SGD) optimizer with a learning rate of 0.1, cross-entropy loss function, and a batch size of 32. Being trained with original CT images, the diagnostic model was able to classify different types of colorectal cancer with an accuracy over 90%.

### F. Evaluation Indexes

To evaluate the performance of the model trained with different datasets, five indexes were used.

#### 1) True Positive Rate (TPR)

TPR refers to the probability of a positive test, conditioned on truly being positive, as shown in (3).

\begin{equation*} TPR=\frac {TP}{TP+FN}\tag{3}\end{equation*}

TP: True Positive FN: False Negative

In this study, we defined it as the probability of correct diagnosis for all Class1 patients.

#### 2) Specificity (Spe)

Spe refers to the probability of a negative test, conditioned on truly being negative, as shown in (4).

\begin{equation*} Spe=\frac {TN}{TN+FP}\tag{4}\end{equation*}

TN: True Negative FP: False Positive

In this study, we defined it as the probability of correct diagnosis for all Class2 patients.

#### 3) False Positive Rate (FPR)

FPR is the proportion of all negatives that still yield positive test outcomes, as shown in (5).

\begin{equation*} FPR=\frac {FP}{FP+TN}\tag{5}\end{equation*}

In this study, we defined it as the probability of actually being Class2 but diagnosed as Class1 patients.

#### 4) False Negative Rate (FNR)

FNR is the proportion of positives which yield negative test outcomes with the test, as shown in (6).

\begin{equation*} FNR=\frac {FN}{TP+FN}\tag{6}\end{equation*}

In this study, we defined it as the probability of actually being Class1 but diagnosed as Class2 patients.

#### 5) Accuracy

Accuracy is the proportion of correct predictions (both true positives and true negatives) among the total number of cases examined, as shown in (7).

\begin{equation*} Accuracy=\frac {TP+TN}{TP+TN+FP+FN}\tag{7}\end{equation*}

In this study, we defined it as the probability of correct diagnosis for all patients.

### G. Workflow

**FIGURE 3.**
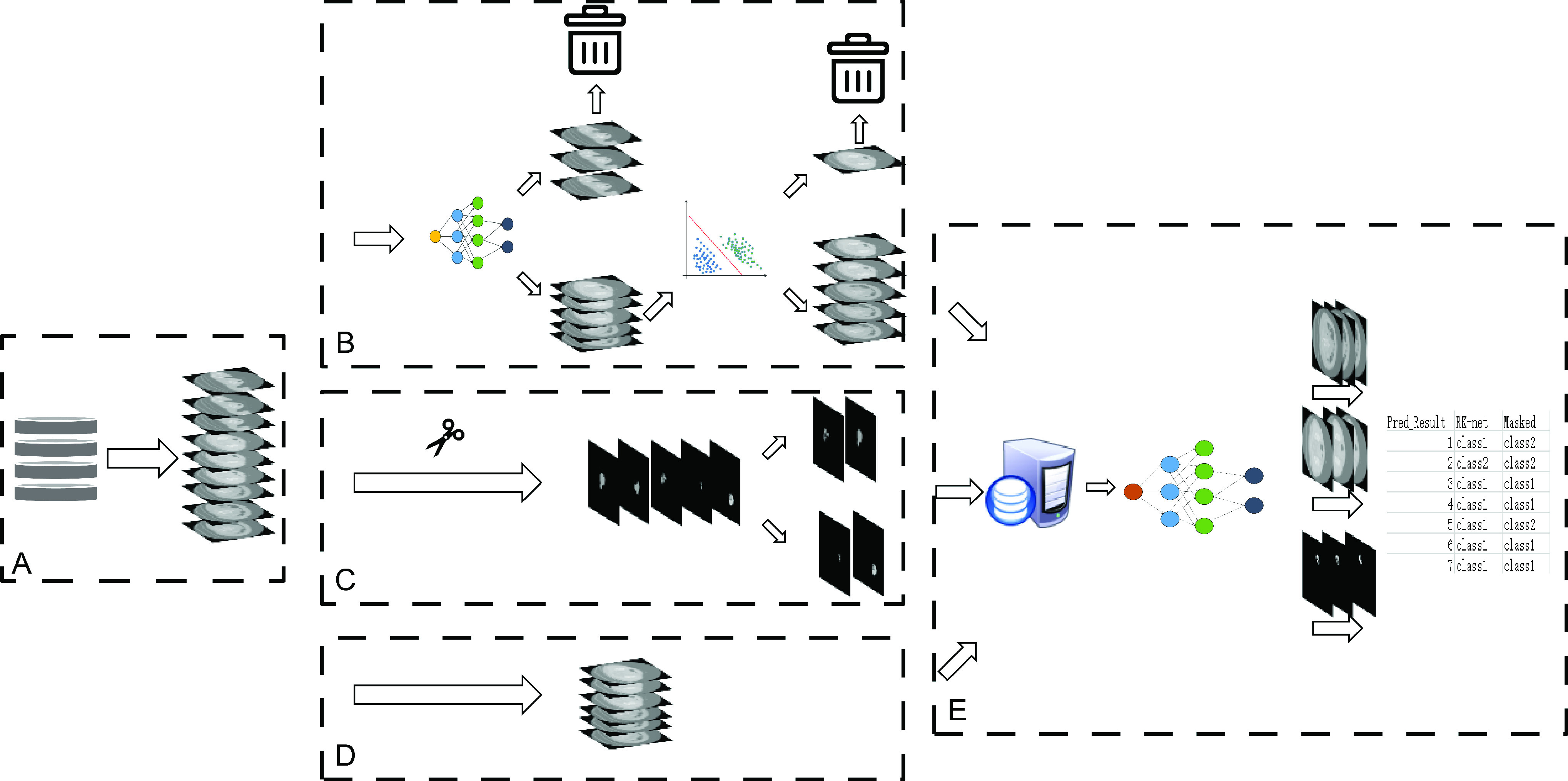
The workflow of study. (A) Image conversion. (B) RK-net process. (C) Manual annotation process. (D) Manual screening process. (E) Training and testing of CRC diagnostic model.

There were three groups of data imported to the CRC diagnostic model on the same platform as described above. CT images were converted to readable formats by programme packages and saved as PNG files. The time costs of data processing and model training were recorded, and the CRC model performances were verified. The workflow of model training and testing is presented in Fig. 3.

## III. Results and Discusions

**FIGURE 4.**
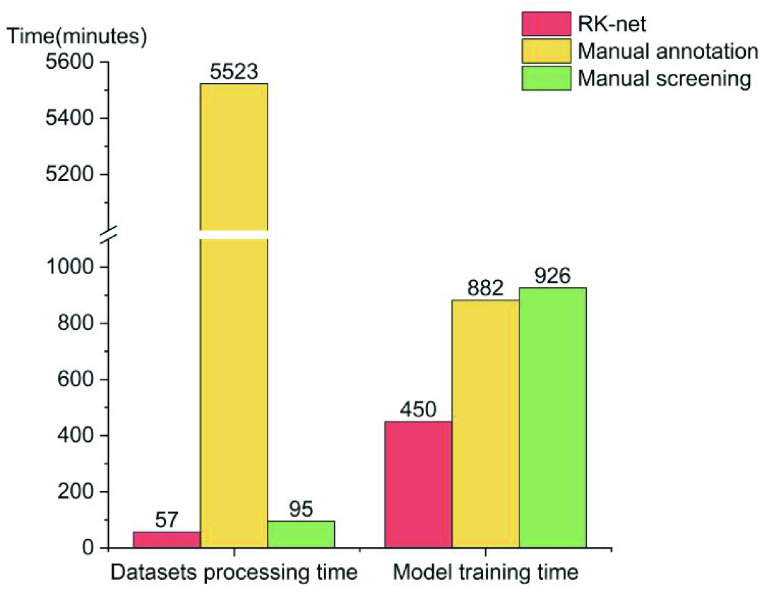
Time cost for data processing and CRC diagnostic model training.

RK-net economized in the time cost for data processing as well as the diagnostic model training (Fig 4). Manual annotation consumed over 100 times the time-cost of the RK-net method for data processing. Moreover, compared with the other methods, it was two times faster to train the diagnostic model with RK-net-processed images.

**FIGURE 5.**
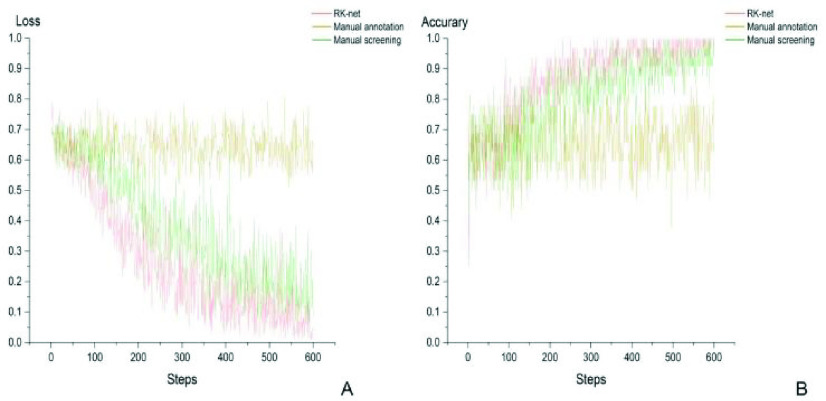
Training loss and accuracy of CRC diagnostic model with different datasets. (A) Model training loss. (B) Model training accuracy.

The DL model for CRC diagnosis performed well in the training process with RK-net refined data, as shown in Fig 5. The training loss decreased rapidly to 0.15 after 400 steps with RK-net, while model training with data from manual screening had a similar trend but a slightly higher loss in the end. The accuracy increased simultaneously with the decrease of training loss, achieving over 0.9 after 500 steps with RK-net or manual screening. However, using the annotated data, the loss fluctuated between 0.6 and 0.8, when the accuracy persisted at a level of 0.6 throughout the training.

The performance of the DL model for CRC diagnosis on testing datasets corresponded to the training process (TABLE 1). The DL model established by the RK-net processed data achieved an accuracy of 0.95 on the testing dataset, while the accuracy of the DL model with manual screening and manual annotation was found to be 0.93 and 0.72, respectively.

**TABLE 1:** The performance of CRC diagnostic model with different datasets

Model	TPR	Spe	FPR	FNR	Accuracy
RM	0.9333	0.9667	0.0333	0.0667	0.9500
AM	0.6667	0.7667	0.2333	0.3333	0.7167
SM	0.9000	0.9667	0.0333	0.0100	0.9333

RM, model with RK-net-processed data. AM, model with manually annotated data. SM, model with manually screened data.

The present study showed the efficacy of RK-net in the optimization of a deep neural network. The proposed method streamlined the original data with the elimination of irrelevant images. The preserved computing resources accelerated the subsequent model training with a high accuracy achievement. Target labeling is often used to outline the ROIs for explicit data input [21]. Manual delineation is usually painstaking and subjective, and useful information could be lost after the deletion of surrounding components [22]. Being trained with incomplete imaging data, a deep neural network may extract incorrect features under certain steps and maintain a low level of accuracy [23].

Considering the development of big data and foundation techniques, advanced models are encouraged to achieve a general recognition of medical images with decreased computing costs [24]. RK-net showed superiority to the existing methods in two aspects. First, it achieved high efficacy in data filtering for complex medical images. The proposed network automatically removed irrelevant images and preserved imaging slices at tumour-level. It avoided the tedious labor of manual screening and annotation. Second, RK-net contributed to the optimization of the subsequent DL model, bringing about lower training costs and better overall performance.

RK-net utilizes the MobileNetV2 network as the low-cost pre-classifier. Owing to the inverted residual with linear bottleneck structure, MobileNetV2 reduces the amount of calculation through lightweight depthwise convolution. This special CNN module achieves memory-efficient inference. It could be readily implemented in Python framework [18]. Combined with the K-means unsupervised classifier, the network could be deployed on a normal server platform without GPU acceleration. The network is also easy to package as a user-friendly tool.

Despite these advantages, RK-net has several limitations as well. First, the composite network relies on pre-trained models with mixed medical images from a wide spectrum of diseases. It is noted that some newly designed algorithms excel in the image classification [25]. Therefore, it is necessary to update the functional modules to enhance the network’s capability. Second, the composite network can only process radiologic images, which could be flawed in the constitution of a complicated model. Future improvements in the generality may realize multimodal data fusion and processing. Last but not least, RK-net needs further validations in different datasets and algorithms.

## IV. Conclusion

In this study, we presented a composite network RK-net that combined deep learning and unsupervised learning algorithms to refine radiological images. RK-net showed efficiency in the elimination of confusing images unrelated to colorectal cancer. The quality control of imaging data was therefore simplified by averting uncontrollable influence associated with human factors. Moreover, RK-net not only decreased the intensive workload of manual screening and annotation, but also improved the performance of deep neural networks from the foundation. This novel algorithm could be a promising method for automatic refinement of medical images in large scale, and assist in the further construction of deep neural networks.

## Supplementary Materials

Supplementary materials
