# Supplementary material for: Unsupervised Learning Composite Network to Reduce Training Cost of Deep Learning Model for Colorectal Cancer Diagnosis
Source: IEEE J Transl Eng Health Med. 2022 Nov 21;11:54–9. doi: 10.1109/JTEHM.2022.3224021 (PMC9762730; doi:10.1109/JTEHM.2022.3224021)
Supplement: Supplementary materials [file supp1-3224021.pdf]

I. Supplementary

The CRC diagnostic model mentioned in the article is derived from an unpublished research : We designed the CRC diagnostic model according to our private database from the Sixth Affiliated Hospital of Sun Yat-sen University (SAH-SYSU), Guangzhou, China, a national high-volume colorectal cancer institution. Our private dataset adopted the data processing method similar to the native data group in this paper. This model was developed based on ResNet-101 algorithm. Our model performance on private test dataset as shown in Fig.1. and TABLE I. The model was able to classify different types of colorectal cancer with accuracy over 90%.

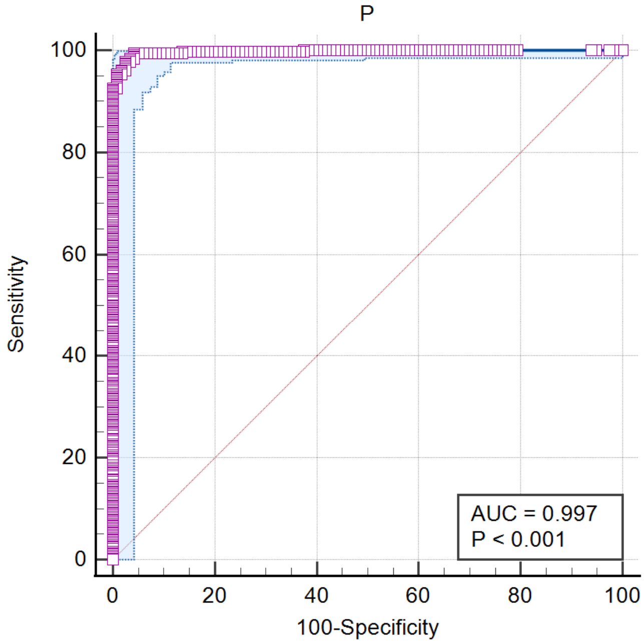

Fig.1. CRC diagnostic model ROC-curve

Area under the ROC curve (AUC)	0.997
Standard Error [a]	0.00141
95% Confidence interval [b]	0.987 to 1.000
z statistic	352.118
Significance level P (Area=0.5)	<0.0001

a. DeLong et al., 1988
b. Binomial exact

TABLE I CRC diagnostic model performance
